# Homozygous *PIGT* Mutation Lead to Multiple Congenital Anomalies-Hypotonia Seizures Syndrome 3

**DOI:** 10.3389/fgene.2018.00153

**Published:** 2018-05-08

**Authors:** Li Yang, Jing Peng, Xiao-Meng Yin, Nan Pang, Chen Chen, Teng-Hui Wu, Xiao-Min Zou, Fei Yin

**Affiliations:** ^1^Department of Pediatrics, XiangYa Hospital, Central South University, Changsha, China; ^2^Department of Neurology, Xiangya Hospital, Central South University, Changsha, China; ^3^Hunan Intellectual and Developmental Disabilities Research Center, Changsha, China

**Keywords:** epilepsy, global developmental delay, GPI-anchored proteins, hypotonia, MCAHS3, multi-malformations, PIGT

## Abstract

*PIGT* encodes a subunit of the glycosylphosphatidylinositol transamidase complex, which catalyzes the attachment of proteins to GPI-anchors. A homozygous *PIGT* variant c.550G>A (p. E184K) in a Chinese boy with multiple malformations, hypotonia, seizure and profound development delay was identified by panel sequencing. Pathogenicity of the variant was confirmed by flow cytometry. The expression of CD16 and CD24 of this proband reduced to 16.92 and 22.16% compare with normal control respectively while which of his parents and sister were normal. This mutation raised the mRNA level on the peripheral blood mono nuclear cells of this patient. This study expanded the variant spectrum of MCAHS3, and CD16 could be an effective marker to evaluate the pathogenicity of *PIGT* mutation.

## Introduction

Multiple Congenital Anomalies Hypotonia-Seizure Syndrome (MCAHS3) is a rare and complicated condition which leads to multiple system dysfunction and is caused by biallelic mutations in PIGT (Kvarnung et al., 2013). The *PIGT* gene (OMIM: 610272) encodes the (GPI) transamidase component of the phosphatidylinositol-glycan biosynthesis class T(PIG-T) enzyme in humans. PIG-T is involved in GPI biosynthesis (Ohishi et al., 2003). GPI-anchors are glycolipids found on membrane of diverse cells that help proteins anchoring to the cell surface. PIG-T mediates GPI anchoring by catalyzing the transfer of fully assembled GPI units to proteins (Ohishi et al., 2003).

Till now, only 12 patients of MCAHS3 were reported. Here, we described a novel variant of *PIGT* in the first case of MCAHS3 among Chinese population, summarized the clinical features of the related genes deficiency in the GPI biosynthesis pathway.

## Methods

### Clinical data collection

The clinical materials were collected by trained pediatric neurologists in the Xiangya Hospital of Central South University, China. The research protocol has been approved by the ethics committee of Xiangya Hospital of Central South University. An informed consent for the gene sequencing and the publication of the results and photos of the patient was signed by the parents, in accordance with the Declaration of Helsinki.

### Panel sequencing

The genomic DNA was sheared by sonication, and then hybridized with a NimbleGen probe (Roche Diagnostic, Switzerland) capture array. The array covered approximately 4000 genes (In house database, Joy Orient, China) of Mendelian inheritance diseases from the Online Mendelian Inheritance in Man (OMIM) database (Joy Orient, China). The libraries were first tested for enrichment by quantitative polymerase chain reaction (qPCR) and for size distribution and concentration using the Agilent Bioanalyzer 2100 (Agilent Technologies, USA). The samples were then sequenced on an Illumina HiSeq 2500 (Illumina, Inc., San Diego, CA, USA). After data filtering at and functional annotation, candidate variants will be confirmed by Sanger sequencing, family members were also examined for the variants.

### Flow cytometry

Flow cytometry was performed with peripheral blood cells collected from the proband, the parents and 3 normal controls. Blood cells from the patient were stained with the following lineage makers and antibodies: mouse anti-CD59(5H8), CD16(3G8), CD24(ML5) antibodies (BioLegend, San Diego, CA, USA) and Alexa 488–fluorochrome-conjugated aerolysin (FLAER; CEDARLANE, Canada), which specifically binds to GPI-anchors. Cells were analyzed by flow cytometry (Canton II; BD Sciences, NY, USA) and FlowJo software (FlowJo, LLC, OR, USA).

### Quantitative real-time PCR

Total RNA was extracted from patient and control cell lines using the RNeasy Mini Kit (QIAGEN), and cDNA was synthesized using the iScript kit (BioRad Laboratories) per the manufacturer protocols. Control reactions were run in parallel without reverse transcriptase. cDNA was amplified using gene-specific primers and iQ SYBR Green Supermix using the following conditions: an initial denaturing steps of 95°C for 10 min, followed by 40 cycles of 95°C for 15 s, 60°C for 10 s, and a final melting curve generated in increments of 0.5°C per plate read, using a CFX96 Touch Real-time PCR Detection System (BioRad Laboratories). Gene expression was quantified using the Ct method with the CFX Manager software (BioRad Laboratories) and all data were corrected against human-actin as an internal control. Primers: human-actin: Fwd 5′ GGCATGGGTCAGAAGGATT 3′ and Rev 5′ TGGTGCCAGATTTTCTCCA 3′; PIGT: Fwd 5′ CTGCGCCTCTCAACTTCA 3′ and Rev 5′ GTGGTAGCTGGTGTGGAACA 3′. Experiments were repeated with 3 technical replicates.

## Results

### Clinical findings

**Figure 1:**
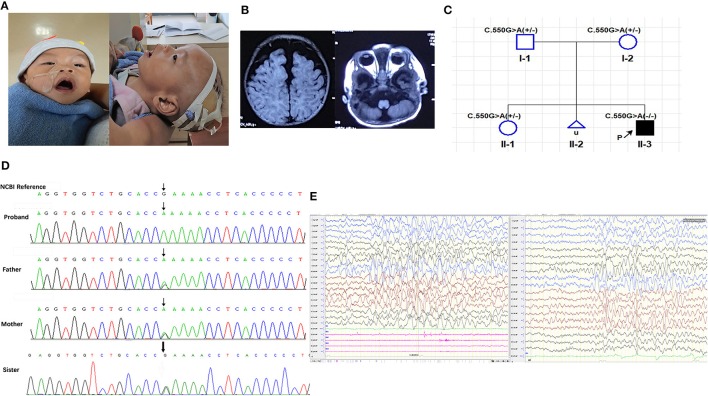
**(A)** Photographs taken at 10 months-old; **(B)** T1 weighted cranial magnetic resonance imaging; **(C)** Family pedigree of segregation of the PIGT c.550G>A; **(D)** Sanger sequencing of the proband and his parents and old sister; **(E)** EEG of the proband at 10 months-old. **(A)** Shown the high forehead with frontal bossing, narrow bitemporal, depressed nasal bridge, tented lip, wide and opening mouth, high arched palate, low ear set of the proband. **(B)** Widen subarachnoid and cerebellar atrophy especially in vermis. **(C)** The *PIGT* c.550G>A segregated with the clinical syndrome, in which the proband is homozygous recessive and his asymptomatic parents and healthy old sister are heterozygous; **(D)** Chromatograms of the Sanger sequencing, showing the homozygous *PIGT* c.550G>A variant in the proband and heterozygous variants in his parents and old sister. **(E)** (Left) Frequent myoclonia on the waking stage; (Right) Numerous of slow waves and slow-spike waves in bilateral hemisphere, especially in posterior head.

The proband, a 10-months-old Chinese boy, the second offspring of a healthy non-consanguineous couple, came to our clinic with a main complaint of “Repeated seizures for 9 months.” He born uncomplicatedly except excessive amniotic fluid, with a birth weight of 3.7 kg and a height of 50 cm. He was hospitalized in the local hospital due to dyspnea shortly after born. During the first hospitalization, he suffered a trunk and limb chattering, no eye gazing nor unconsciousness, remitted after a few seconds. Unfortunately, no EEG was addressed at that time. At 6 months old, the events relapsed with a body temperature of 38°C, accompanied with irritableness, followed by head rotating rightward, eyes staring, limbs jittering, and distortion of commissure rightward for 2–3 min, then returned to consciousness. The EEG showed absent spindle waves, normal background without epileptic discharges. No antiepileptic therapy was addressed until the proband transferred to our hospital, the EEG showed background wave slowing, bilateral slow waves discharging, especially in the posterior head, frequent myoclonus with concurrent EEG changes during waking stage (Figure 1E), and then levetiracetam solution was administrated to him at a maximum dose of 1 ml twice daily (28.6 mg/kg. d). The epileptic attack was controlled after this therapy. However, he still suffered from recurrent respiratory infections, and was hospitalized several times due to severe pneumonia requiring oxygen therapy or continuous positive airway pressure support.

The developmental delay was noticed after birth. He suffered feeding difficulty, requiring stomach tube support, could not raise his head at 10 months old nor sit up by himself. At 7 months of age, he was babbling unconsciously with a hoarse voice, and he was identified as throat-trachea-bronchial softening by bronchoscopy at 5 months of age. Besides, poor head controlling was noticed, resulting in forward or backward head tilting (Supplementary Video S1).

Both his parents are asymptomatic, as well as his elder sister. An unborn sibling was artificially aborted (Figure 1C) due to cerebellar aplasia diagnosed by fetal ultrasonography.

Physical examination showed he had a normal head circumference of 44.5 cm (48.5 percentile), high forehead, frontal bossing, narrow bitemporal, big eyes with slight orbital depression, esotropia, depressed nasal bridge, long philtrum, high palatine arch, wide and opening mouth, low auricular position (Figure 1A), hypotonia (Supplementary Video S1) and lower limb hyperreflexia.

The serum bio-chemical findings were both normal including liver and kidney function, blood glucose, myocardial enzymes, electrolytes, and alkaline phosphatase. Both urine and blood organic acid and amino acids were unremarkable.

Cranial magnetic resonance imaging (MRI) showed an external hydrocephalus and a widening of the subarachnoid and frontal temporal spaces, particularly frontally, suggesting cortical hypoplasia and cerebellar vermis dysplasia (Figure 1B).

### Panel sequencing

The panel sequencing revealed a homozygous missense *PIGT* mutation, c.550G>A(exon 4), p.E184K(rs774753616), NM_015937.5 lies in the adjacent codon to a previously reported pathogenic variant p.(T183P), (Kvarnung et al., 2013). This variant was confirmed by Sanger sequencing, both his asymptomatic parents, as well as his healthy old sister are heterozygous (Figures 1C,D). This variant was predicted to be likely pathogenic (Proven, Intervar, Polyphen 2, Sift, Mutationtaster, M-CAP, REVEL). The minor allele frequency (MAF) of this variant is 0.00001626 and 0.0000 in East Asian people (gnom AD). Basic on the clinical manifestations of the proband and the principle of familial co-segregation, variants identified by panel sequencing but consider as non-pathogenic were listed on Table 2.

### Flow cytometry

**Figure 2:**
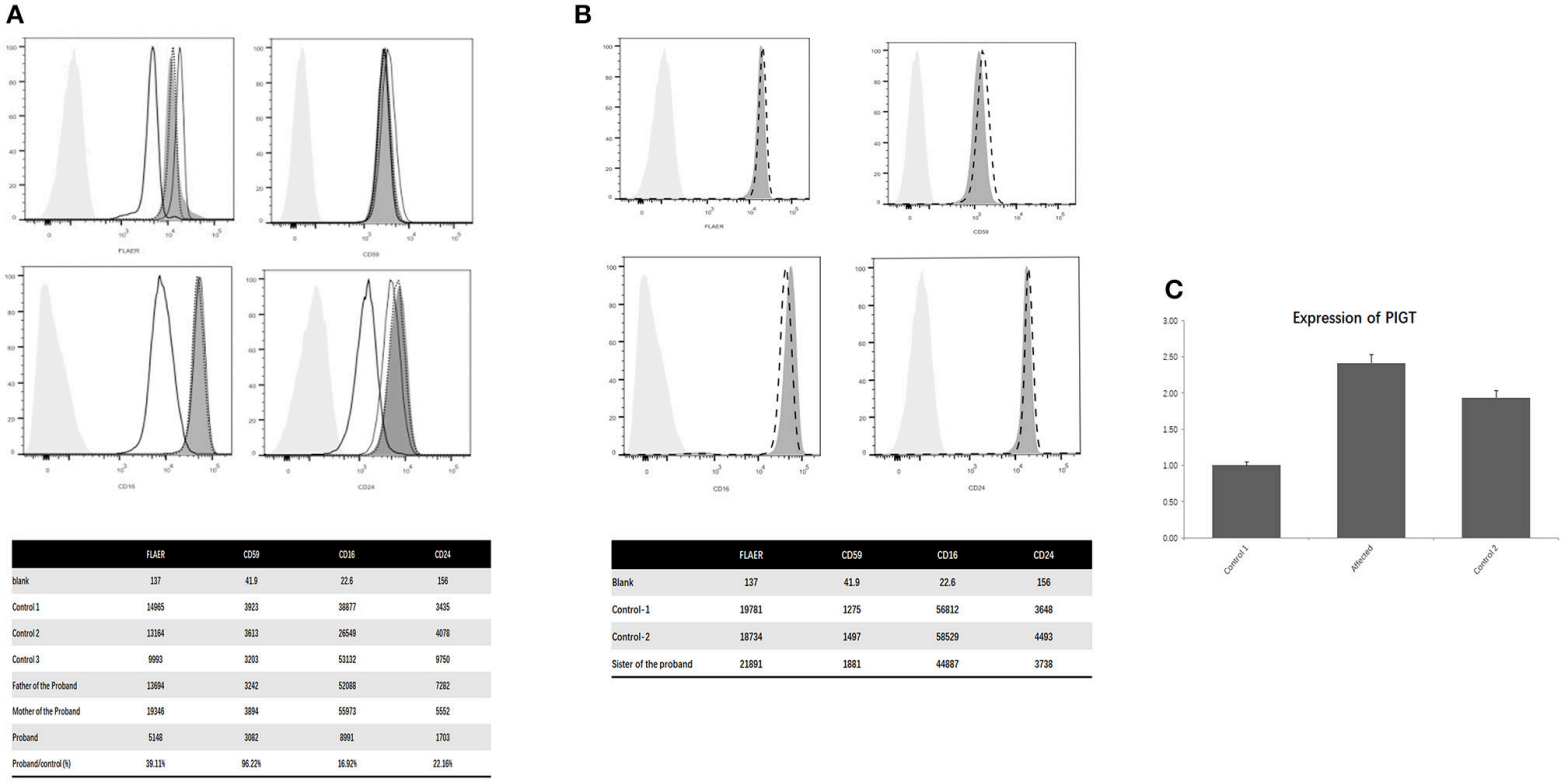
**(A)** Flow cytometry of the proband, parents and controls; **(B)** Flow cytometry of the old sister of the proband and the controls; **(C)** PIGT mRNA expression on PBMCs. **(A)** The abscissa indicates the intensity of the fluorescence, the ordinate indicates the number of cells, the light gray shade represents the blank control, the dark gray shade represents the normal control, the black thick line represents the patient, the black fine line represents his mother, the black dotted curve represents his father. Shown that the expression of total GPI anchoring molecules in this patient was reduced to 39.11% of the normal controls. The expression of CD59 of the proband was similar with the normal controls. The expression of CD16 was reduced to 16.92% of the normal control and the expression of CD24 was reduced to 22.16%. Meanwhile, the abundance of the same proteins in the leucocytes of his parents was normal. **(B)** Shown that the level of CD16, CD24 and CD59 of the old sister (the dotted line) of the proband had no significant difference with the normal controls (The dark gray shades). **(C)** The level of PIGT mRNA on PBMCs of the proband was significantly higher than the two healthy controls **(C)**.

Flow cytometry showed that the expression of total GPI-anchoring molecules of the proband was reduced to 39.11% compared to the normal controls. The expression of CD59 of the proband was 96.22% compared to the normal controls. The expression of CD16 of the proband was reduced to 16.92% of the normal control, and the expression of CD24 of the proband was reduced to 22.16% (Figure 2A). The abundance of the same proteins of his parents, as well as his old sister (Figures 2A,B).

### Quantitative real-time PCR

PIGT mRNA levels was evaluated by performing quantitative real-time PCR analysis on peripheral blood mononuclear cells (PBMCs) from the proband and age-matched controls. The result of QPCR indicated that the level of PIGT mRNA on PBMCs of the proband increased significantly (Figure 2C).

## Discussion

More than 100 proteins in mammalian cells are translated and modified at the C end by adding glycosylphosphatidylinositol (GPI), then anchored on the cell surface through the lipid moiety. GPI anchored proteins have many functions including cell recognition, growth, differentiation and programmed cell death (Cinek and Horejsi, 1992). During the GPI attachment process, GPI transamidase forms a carbonyl intermediate with the precursor protein, and due to the absence of *PIGT*, the carbonyl intermediate cannot be generated. Moreover, lacking of PIGT results in the association between GAA1 and GPI8–two components required in mammalian GPI transamidases declining, also leads to decreased stability of GPI complex. *PIGT* mutants may not be able to correctly form the GPI transamidase complex, leading to a loss of GPI transamidase activity and expressive reduction of GPI-anchored proteins in the cellular surface (Ohishi et al., 2003).

The GPI biosynthetic pathway of mammals includes 11 upstream steps and 4 downstream steps, over 20 genes were involved. Among these genes, *PIGY, PIGW, PIGG, PIGM, PIGV, PIGN, PIGL, PIGA, PIGO, PIGT, PIGC, PIGQ, PIGP, PGAP1, PGAP2, and PGAP3* mutations were reported relating to human genetic diseases (Freeze et al., 2014; Supplementary Table 1). Despite their different functions during GPI biosynthesis, disorders related to these mutations share the following clinical features: multiple facial, head and skeletal anomalies, epileptic seizures, hypotonia, with or without congenital heart diseases, poor feeding, genitourinary system abnormalities like paroxysmal nocturnal haemoglobinuria and prenatal abnormality such as polyhydramnios (Supplementary Table 2).

The major symptoms of *PIGT* mutant were similar with other phenotypes of GPI biosynthesis gene family deficiency, however, there were still some slight differences. None of the patients with *PIGT* related MCAHS3 was reported to have a nail, skin or hair anomaly while most of the other genes did, on the contrary, only patients of *PIGT* related MCAHS3 was reported to have hypercalciuria (Kvarnung et al., 2013; Nakashima et al., 2014; Lam et al., 2015; Skauli et al., 2016; Pagnamenta et al., 2017). Additionally, the highest expression level of PIGT is in the central nervous system, which may explicate the most severe neurological symptoms of *PIGT* mutant. Moreover, homozygous null *PIGT* mutation in mice was proved to be lethal, also proved its severity (Blake et al., 2017).

A recognizable facial dysmorphic pattern in MCAHS3 includes a high forehead, frontal bossing, bitemporal narrowing, a short nose with depressed nasal bridge, and a wide, opening mouth, tented lips, high-arched palate, low auricular position (Table 1). Cardiac abnormalities were identified in only three patients of all reports, and most of them were mild (Kvarnung et al., 2013; Nakashima et al., 2014; Lam et al., 2015; Skauli et al., 2016; Pagnamenta et al., 2017), indicating that cardiac anomaly is not the core feature of this condition. According to previous studies, seven of the patients with MCAHS3 had low serum alkaline phosphatase. Some of these reports proposed that hypophosphatemia may be the cause of skeletal abnormalities as well as the epileptic seizures (Kvarnung et al., 2013; Nakashima et al., 2014; Pagnamenta et al., 2017). However, normal levels of serum alkaline phosphatase were detected in both our proband and the patients reported by Lam (Lam et al., 2015) and Skauli (Skauli et al., 2016), which may indicate that hypophosphatemia cannot entirely explain the above symptoms. Febrile seizure was observed in half of the *PIGT* mutants, mostly between 4 and 6 months age, followed by clusters of refractory seizures. Repeated MRI examinations in these patients revealed progressive cerebellum atrophying, indicating neurodegeneration, and this might interpret the pathogenies of refractory seizure. Of note, 6 of the reported patients presented with feeding difficulties (Table 1). Another noteworthy point is, no *PIGT* mutant except our proband has been reported with airway softening, leading to expectoration difficulty, which may aggravate the recurrent respiratory infection. Therefore, in those patients with *PIGT* mutation and recurrent respiratory infection, bronchoscope should be considered. Although the pathogenicity is not clear, it is speculated to be related to osteal or cartilaginous metabolic anomalies. However, since this proband is still in infancy, more follow-up is needed, and more cases should be collected to identify more precise clinical features.

**Table 1:** Summary of the clinical features in patients with *PIGT* related MCAHS3 reported to date.

**References**	**Yang et al**.	**Pagnamenta et al**.			**Skauli et al**.		**Lam et al**.		**Nakashima et al**.	**Kvarnung et al**.			
**Patients**	**1**	**1**	**2**	**3**	**1**	**2**	**1**	**2**	**1**	**1**	**2**	**3**	**4**
*PIGT* variants	c.550G>A; p. E184K	c.1582G4A; p.(V528M)	c.709G4C; p.E237Q	c.709G4C; p.E237Q	c.1079G>T; p.G360V	c.1079G>T; p.G360V	c.918dupC; p.V307Rfs*13	c.918dupC; p.V307Rfs*13	c.250G>T; p.E84*	c.547A>C; p.T183P			
		c.1730dupC; p.L578fs*35					c.1342C>T; p.R488W	c.1342C>T; p.R488W	c.1342C>T; p.R488W	
Ethnic	Chinese	Caucasian	Afghanistan	Afghanistan	Somalian	Somalian	Caucasian mother/African American father	Caucasian mother/African American father	N/A	N/A	N\A	N/A	N/A
Gender	M	M	M	M	M	M	F	M	F	F	F	F	F
GA weeks	40	N/A	N/A	N/A	40	40	<32	<32	40	40	39	37	37
Frontal bossing	(+)	(+)	N/A	N/A	(+)	(+)	(+)	(+)	(+)	(+)	(+)	(+)	(+)
High forehead	(+)	(+)	N/A	N/A	(+)	(+)	(+)	(+)	(+)	(+)	(+)	(+)	(+)
Bitemporal narrowing	(+)	(+)	N/A	N/A	(+)	(+)	(+)	(+)	(+)	(+)	(+)	(+)	(+)
Impaired vision	N/A	N/A	N/A	N/A	(+)	(+)	(+)	(+)	N/A	(+)	(+)	(+)	(+)
Strabismus	(+)	N/A	N/A	N/A	(+)	(+)	(+)	(+)	N/A	(+)	(+)	(+)	(+)
Nystagmus	(−)	N/A	(+)	N/A	(+)	(+)	(+)	(+)	N/A	(+)	(+)	(+)	(+)
Hear defect	(−)	N/A	N/A	N/A	(−)	(−)	(+)	(+)	N/A	N/A	N/A	N/A	N/A
Depressed nasal bridge	(+)	(+)	N/A	N/A	(+)	(+)	(+)	(+)	(+)	(+)	(+)	(+)	(+)
Open mouth	(+)	(+)	N/A	N/A	(+)	(+)	N/A	N/A	N/A	N/A	N/A	N/A	N/A
Tented lips	(+)	(+)	N/A	N/A	(+)	N/A	N/A	N/A	N/A	N/A	N/A	N/A	N/A
Low ear-sets	(+)	(+)	N/A	N/A	N/A	N/A	N/A	N/A	N/A	N/A	N/A	N/A	N/A
High-arched palate	(+)	(−)	N/A	N/A	(+)	(+)	N/A	N/A	N/A	N/A	N/A	N/A	N/A
Tooth abnormalities	(+)	(+)	N/A	N/A	(+)	(+)	(−)	(−)	N/A	(+)	(+)	(+)	(+)
Restrictive cardiomyopathy	(−)	N/A	N/A	N/A	(−)	(−)	N/A	N/A	N/A	(−)	(+)	N/A	(−)
Patent ductus arteriosus	(−)	N/A	N/A	N/A	(−)	(−)	(−)	(−)	(+)	(+)	(−)	(−)	(−)
Inverted nipples	(−)	N/A	N/A	N/A	(+)	(+)	N/A	N/A	N/A	(−)	(−)	(+)	(+)
Osteopenia	(−)	N/A	N/A	N/A	(−)	(−)	(+)	N/A	N/A	N/A	N/A	N/A	N/A
Osteoporosis	(−)	N/A	N/A	N/A	(−)	(−)	N/A	N/A	(+)	N/A	N/A	N/A	N/A
Delayed bone age	N/A	N/A	N/A	N/A	(−)	N/A	N/A	N/A	N/A	(+)	(+)	(+)	(+)
Scoliosis	(−)	N/A	N/A	N/A	N/A	N/A	(−)	(−)	N/A	(+)	(+)	(−)	(−)
Hypotonia	(+)	N/A	N/A	N/A	(+)	(+)	(+)	(+)	(+)	(+)	(+)	(+)	(+)
Severe Motor and intellectual disability	(+)	(+)	(+)	(+)	(+)	(+)	(+)	(+)	(+)	(+)	(+)	(+)	(+)
Feeding difficulty	(+)	N/A	(+)	N/A	(+)	N/A	(+)	N/A	(+)	N/A	N/A	N/A	N/A
Febrile sensitive seizures	(+)	(+)	N/A	N/A	(+)	(+)	(+)	N/A	(+)	N/A	N/A	N/A	N/A
Seizures	(+)	(+)	(+)	(+)	(+)	(+)	(+)	(+)	(+)	(+)	(+)	(+)	(+)
Refractory seizures	N/A	N/A	(+)	(+)	(+)	(+)	(+)	(+)	(+)	N/A	N/A	N/A	N/A
EEG abnormalities	(+)	(+)	(+)	(+)	(+)	(+)	(+)	(+)	(+)	(+)	(+)	(+)	(+)
Airway softening	(+)	N/A	N/A	N/A	N/A	N/A	N/A	N/A	N/A	N/A	N/A	N/A	N/A
Recurrent respiratory infections	(+)	N/A	N/A	N/A	N/A	N/A	N/A	N/A	(+)	N/A	N/A	N/A	N/A
Microcephaly	(−)	N/A	(+)	(+)	N/A	N/A	N/A	N/A	N/A	N/A	N/A	N/A	N/A
Cerebral atrophy	(+)	N/A	N/A	N/A	(+)	(+)	(+)	(+)	(+)	(−)	(−)	(+)	(+)
Ataxia	(+)	(+)	N/A	N/A	(+)	(+)	N/A	N/A	N/A	N/A	N/A	N/A	N/A
Cerebellar hypoplasia	(+)	(+)	N/A	N/A	(+)	(+)	(+)	(+)	(+)	(−)	(−)	(+)	(+)
Decreased alkaline phosphatase	(−)	(+)	(+)	N/A	(−)	(−)	(−)	(−)	(+)	(+)	(+)	(+)	(+)
Nephrolithiasis	(−)	(+)	N/A	N/A	(−)	(−)	N/A	N/A	N/A	N/A	N/A	N/A	N/A
CD16 level decrease	(+)	(+)	N/A	N/A	(+)	N/A	(+)	(+)	(+)	N/A	N/A	N/A	N/A
CD24 level decrease	(+)	N/A	N/A	N/A	(+)	N/A	N/A	N/A	(−)	N/A	N/A	N/A	N/A
CD59 level decrease	(−)	(+)	(+)	(+)	N/A	N/A	N/A	N/A	N/A	N/A	N/A	N/A	N/A
CD48 Level decrease	N/A	N/A	N/A	N/A	(+)	N/A	N/A	N/A	N/A	N/A	N/A	N/A	N/A
CD14 Level decrease	N/A	N/A	N/A	N/A	(+)	N/A	N/A	N/A	N/A	N/A	N/A	N/A	N/A

*M: Male; F: Female; (+): Features presented; (−): Features absented; N/A: Not applicable or not determined*.

**Table 2:** The non-pathogenic variants identified by panel sequencing.

**Gene (Chromosome)**	**Nucleotide alternation (exon)**	**Amino acid alternation**	**MAF**	**Genotype of the proband(male)**	**Genotype of the father (Normal phenotype)**	**Genotype of the mother (Normal phenotype)**	**Related disease (Hereditary mode)**
GALC (chr14)	c.163T>C (exon 1)	p.F55L(NM_000153)	Null	Het	Het	WT	Krabbe disease (OMIM:245200, AR)
IFIH1 (chr2)	c.2115A>C (exon 11)	p.R705S(NM_022168)	<0.0098	Het	Het	WT	Aicardi-Goutieres syndrome 7(OMIM:615486, AD)
MTFMT (chr15)	c.797G>A (exon 6)	p.R266H(NM_139242)	<0.003	Het	WT	Het	Combined oxidative phosphorylation deficiency 15(OMIM:614947, AR)
NEB (chr2)	c.19295A>G (exon 124)	p.Q6432R(NM_001271208)	<0.015	Het	Het	WT	Nemaline myopathy 2(OMIM:256030, AR)
PIEZ02 (chr18)	C.7011-3C>A (IVS 44)	_(NM_022068)	<0.017	Het	Het	WT	Marden-Walker syndrome (OMIM:248700, AD)
TINF2 (chr14)	c.573A>T (exon 5)	p.Q191H(NM_012461)	Null	Het	WT	Het	Revesz syndrome (OMIM:268130, AD)

The *PIGT* variant cause significant reductions in the level of GPI-anchored proteins, especially CD16, which has been observed in 6 of the 12 reported patients (Figure 2A,B, Table 1). CD 16 is found on the surface of various sorts of cells including natural killer cells, neutrophil polymorphonuclear leukocytes, monocytes and macrophages (Zeng et al., 2016). Reduction of CD16 indicates a severe decline of cell immunity, also might be another etiology of the recurrent respiratory infections of our proband. In this regard, CD 16 degeneration might be a key marker of the evaluation of the pathogenicity of *PIGT* mutation. Interestingly, the diseases caused by mutations in genes involved in GPI biosynthesis pathway present with decreased levels of GPI-anchored proteins, including CD16, CD14, CD18, CD24, CD55, CD59, etc. except *PIGG* mutations (Supplementary Table 2). This makes the GPI-anchored proteins plausible to be the significant biomarkers of the pathogenicity for GPI-anchored protein deficiency. And if applicable, all the above GPI- anchored proteins should be measured in the patients suspected with GPI-deficiency. And future researches on fixing GPI biosynthesis or GPI-Aps anchoring may be a pathway to resolve these disorders. Interestingly, in this case, the mRNA of PIGT on the PMBCs of this proband increased instead of decreasing. This result is similar with the finding of Lam et al. (2015) One potential explication is, since the amino acid change in this patient was glutamate to lysine, lead to a dramatic polarity changing, eventually causing losing of function, therefore, the mRNA increasing may contributed by negative feedback regulation, however, due to the persistence of mutation, the increased expression of mRNA, as well as the protein elevation (Lam et al., 2015), cannot rescue the losing of function. Notably, the histone deacetylase inhibitor-butyrate has been reported to be capable to increase the *PIGM* transcription and GPI expression on cellular surface, and control the intractable seizures of a child with *PIGM* mutation,(Almeida et al., 2007) that might also be a gateway to deal with patient with *PIGT*.

In conclusion, we identified a novel, likely pathogenic homozygous *PIGT* missense variant c.550G > A (p. E184K) in a Chinese Boy with multiple exterior abnormalities and severe multi-neurological disorders by panel sequencing. The pathogenicity of this mutation was proved by flow cytometry, in which the GPI-anchored proteins (CD16, CD24) of this proband decreased significantly. This research expanded the variant spectrum of MCAHS3, and this is the first report of *PIGT* mutant among Chinese population. In patients with multiple exterior abnormalities combined with severe psychomotor retardation/regression, intractable seizures, feeding difficulty and hypotonia, GPI-deficiency should be considered, and CD16 could be an effective marker to evaluate the pathogenicity of *PIGT* mutation.

## Author contributions

LY wrote the manuscript, collected the clinical materials and blood samples of the patients; X-MY performed the cell flowmetry and revised the manuscript; CC and NP referred the patient for study and coordinated the clinical analysis of the patient; T-HW and X-MZ supplemented the supplementary experiments; FY and JP initiated the study, made substantial contribution to conception design and revised the manuscript critically.

### Conflict of interest statement

The authors declare that the research was conducted in the absence of any commercial or financial relationships that could be construed as a potential conflict of interest.
